# Association of inflammation indices with left atrial thrombus in patients with valvular atrial fibrillation

**DOI:** 10.1186/s12872-023-03036-x

**Published:** 2023-01-09

**Authors:** You Zhou, Xuewen Song, Jifang Ma, Xianqing Wang, Haixia Fu

**Affiliations:** 1 Heart Center of Henan Provincial People’s Hospital, Central China Fuwai Hospital, Central China Fuwai Hospital of Zhengzhou University, No.1 Fuwai Avenue, Zhengdong New District, Zhengzhou, 450003 Henan China; 2 Department of Hematology, The Affiliated Cancer Hospital of Zhengzhou University and Henan Cancer Hospital, Hemostasis and Thrombosis Diagnostic Engineering Research Center of Henan Province, Zhengzhou, 450008 China

**Keywords:** Inflammation, Left atrial thrombus, Valvular atrial fibrillation

## Abstract

**Background:**

Inflammation has been implicated in the progressive exacerbation of valvular atrial fibrillation (VAF) and thrombogenesis. This study aimed to analyze the association of systemic inflammation as measured by six indices with left atrial thrombus (LAT) in patients with VAF.

**Methods:**

This comparative cross-sectional analytical study included 434 patients with VAF. Logistic regression analysis was used to assess the predictive value of LAT using six inflammation indices: neutrophil-to-lymphocyte ratio, monocyte-to-lymphocyte ratio (MLR), white blood cell-to-mean platelet volume ratio, neutrophil-to-mean platelet volume ratio, systemic immune inflammation index, and systemic inflammation response index. Receiver operating characteristic curves were plotted, and the area under these curves (AUC) were calculated to evaluate the discriminative ability of the indices.

**Results:**

Transesophageal echocardiography revealed LAT in 143 (32.9%) patients. All six indices reflected a positive correlation with C-reactive protein levels. Multivariate logistic analysis revealed that these indices were independent predictors of LAT, and MLR appeared to perform best (odds ratio 12.006 [95% confidence interval (CI) 3.404–42.347]; *P* < 0.001; AUC 0.639 [95% CI 0.583–0.694]; *P* < 0.001).

**Conclusions:**

Selected inflammatory indices were significantly and independently associated with LAT among patients with VAF.

## Background

Atrial fibrillation (AF) is the most common type of cardiac arrhythmia and its health-related burden continues to be significant worldwide. The estimated prevalence of AF among adults is 2–4%, and it is expected to increase owing to extended longevity [1]. AF has been proved to an important contributor to stroke, resulting in substantial morbidity and mortality. Approximately, 30% of AF patients, who experience stroke, die within 1 year, and 15–30% of stroke survivors remain permanently disabled [2]. AF can be classified into “valvular AF (VAF)” and “nonvalvular AF (NVAF)” [1]. Compared with the latter, the former heralds in greater embolic risk. Patients with VAF who have experienced embolic events have recurrences at a rate of 15–40 events per 100 patient-months, which is the highest rate of thromboembolism ever reported in AF [3]. Notably, VAF related stroke occurs in a much younger population with consequent loss of human power and resultant economic burdens [4].

Inflammatory immune response is a determinant of initiation and progressive exacerbation of VAF. Degenerative remodeling, extensive fibrosis and evidence of ongoing inflammation has been found in the left atria of VAF patients by histopathological studies [5, 6]. It has been well recognized that the left atrial thrombus (LAT) is the primary cause of stroke in AF patients [7]. Chronic systemic inflammation and oxidative injury play a vital role in LAT formation in patients with AF, which may lead to endothelial dysfunction and a hypercoagulable state. Inflammatory biomarkers, such as C-reactive protein (CRP), were proved to be associated with the presence of LAT in patients with AF [8].

Several hematological indices, including neutrophil-to-lymphocyte ratio (NLR), monocyte-to-lymphocyte ratio (MLR), white blood cell-to-mean platelet volume ratio (WMR), neutrophil-to-mean platelet volume ratio (NMR), systemic immune inflammation index (SII), and system inflammation response index (SIRI), are also believed to reflect inflammation [9]. These leukocyte-derived indices integrate information from the innate and adaptive immunity to avoid relying on the absolute value of a single leukocyte subtype caused by infection or dehydration. Evidence from observational studies has demonstrated a significant association between these inflammation indices and the incidence and severity of many cardiovascular diseases, such as heart failure, myocardial infarction, and hypertension [10]. However, to date, no studies have investigated the predictive value of inflammation indices for LAT in VAF patients. The objective of this study was to determine the association of NLR, MLR, WMR, NMR, SII and SIRI with the risk of LAT formation in patients with VAF undergoing transesophageal echocardiography (TEE).

## Methods

### Study population

**Fig. 1:**
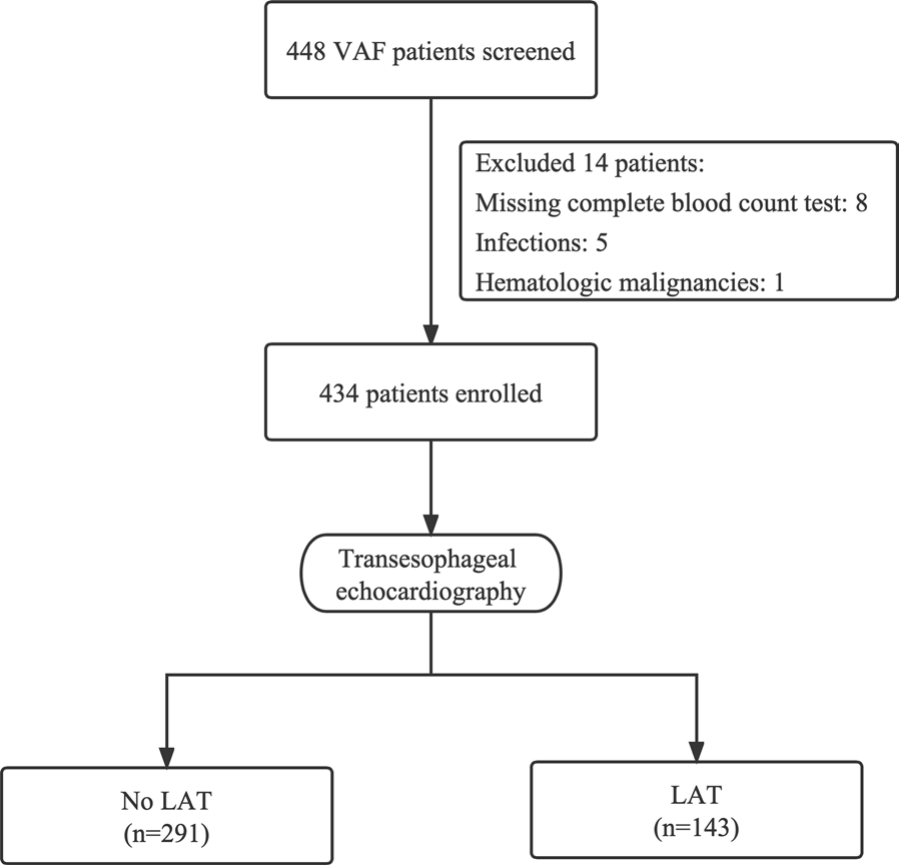
Flow diagram showing screening and recruitment of the study population. *VAF* valvular atrial fibrillation, *LAT* left atrial thrombus

A total of 448 patients with documented VAF who underwent TEE at Henan Provincial People’s Hospital between January 2015 and September 2022 were retrospectively collected. VAF refers to AF patients with moderate/severe mitral stenosis and those with mechanical prosthetic heart valve(s) [1]. First-diagnosed AF was defined as AF not diagnosed previously, irrespective of its duration or the presence/severity of AF-related symptoms [1]. Patients with hematological malignancies, acute and/or chronic infection, or missing complete blood count data were excluded (n = 14). Ultimately, 434 participants were enrolled in the current study (Fig. 1). The study protocol was approved by the Ethics Committee of the Henan Provincial People’s Hospital. The need for informed consent was waived by the Ethics Committee of the Henan Provincial People’s Hospital, because of the retrospective nature of the study.

### Data collection

Patients’ demographic characteristics, including age, gender, comorbidities (coronary heart disease, hypertension, heart failure, type of AF, diabetes, and stroke), echocardiographic parameters, laboratory variables, and medications (oral anticoagulants, beta-blockers, diuretics and renin-angiotensin system inhibitors) were obtained from patients’ medical records before TEE.

### Biochemical analyses

Venous blood samples were collected from each patient after 12 h fasting on the day before TEE. All these tests were performed at the core laboratory of Henan Provincial People’s Hospital using standard techniques. Biochemical parameters were determined by Hitachi 7180 biochemistry autoanalyzer. The Cockcroft-Gault equation was used to estimate the glomerular filtration rate.

### Calculation of inflammation indices

The inflammation indices were determined from the first blood test results after admission. The ratios were calculated using the following equations:

$$\begin{aligned} & {\text{NLR }} = {\text{ total }}\;{\text{number }}\;{\text{of }}\;{\text{neutrophils/total }}\;{\text{number }}\;{\text{of }}\;{\text{lymphocytes}}; \\ & {\text{MLR }} = {\text{ total }}\;{\text{number}}\;{\text{ of }}\;{\text{monocytes/total }}\;{\text{number }}\;{\text{of }}\;{\text{lymphocytes;}} \\ & {\text{WMR}} = {\text{ total}}\;{\text{ number }}\;{\text{of }}\;{\text{white}}\;{\text{ blood }}\;{\text{cell/mean }}\;{\text{platelet }}\;{\text{volume}}; \\ & {\text{NMR}} = {\text{ total }}\;{\text{number }}\;{\text{of }}\;{\text{neutrophils/mean }}\;{\text{platelet}}\;{\text{ volume}}; \\ & {\text{SII}} = \, \left( {{\text{total}}\;{\text{ number}}\;{\text{ of }}\;{\text{neutrophils }} \times {\text{ total }}\;{\text{number }}\;{\text{of }}\;{\text{platelets}}} \right)/{\text{total }}\;{\text{number }}\;{\text{of }}\;{\text{lymphocytes}}; \\ & {\text{SIRI}} = \, \left( {{\text{total }}\;{\text{number }}\;{\text{of }}\;{\text{neutrophils }} \times {\text{ total }}\;{\text{number }}\;{\text{of }}\;{\text{monocytes}}} \right)/{\text{total }}\;{\text{number }}\;{\text{of}}\;{\text{ lymphocytes}}{.} \\ \end{aligned}$$

### Echocardiographic data

TEE was performed by two qualified physicians to detect thrombus formation in the left atrium. LAT was defined as a well-circumscribed, highly reflective mass with a different texture from the atrial wall and uniform consistency [11]. The severity of mitral stenosis was evaluated using the peak and mean gradients obtained at the mitral inflow velocities using continuous wave Doppler ultrasound scanning from the apical view under transthoracic echocardiography before TEE. Mitral valve area was calculated using the pressure half-time method and planimetry of the mitral valve orifice in early diastole from the short-axis view. Patients with a valve area < 1.5 cm^2^ or a mean gradient > 5 mmHg were considered to have moderate/severe mitral stenosis. Left atrial diameter (LAD) and left ventricular ejection fraction (LVEF) were measured from M-mode or 2D view in the parasternal long-axis projection. Discrepancies were resolved through discussions or by an expert physician when needed.

### Statistical analysis

Continuous variables were presented as the means ± standard deviations or medians (inter-quartile ranges). The Student’s *t* tests and Mann–Whitney U tests were used to compare normally and non-normally distributed variables, respectively. Categorical variables in each group, expressed as percentages, were compared using the *χ*^2^ test. Spearman test was used to evaluate the correlation between inflammation indices and CRP levels. Restricted cubic splines were used to assess the dose–response association between inflammation indices and the risk for LAT. Four knots were placed at the 5th, 35th, 65th, and 95th percentiles of inflammation indices. A univariate logistic regression model was used to assess the association between the inflammation indices and LAT, followed by multivariate adjustments. Model 1 was also adjusted for age and gender. Model 2 was additionally adjusted for thrombotic factors (coronary heart diseases, heart failure, hypertension, diabetes, stroke). Model 3 was adjusted for the factors in model 2 in addition to oral anticoagulants, LAD and LVEF. Odds ratios (ORs) and 95% confidence intervals (CIs) were calculated to express the risk. Receiver operating characteristic (ROC) curves were plotted to identify the cut-off values of inflammation indices that could be used to predict LAT. The area under the ROC curves (AUC) were calculated and compared pairwise. A value of *P* < 0.05 was considered significant in all conditions. Statistical analyses were performed using SPSS Statistics 26.0 (SPSS, Chicago, IL, USA) and R 4.1.2 (R Core Team, Vienna, Austria).

## Results

### Baseline characteristics

The mean age of the 434 patients was 56.94 ± 9.12 years, and only 36.2% were men. 32.7% of included patients received oral anticoagulant at admission. LAT was observed in 143 (32.9%) patients. Patients with LAT were more likely to be male, and had heart failure and diabetes, with larger LAD and higher CRP levels, than those without LAT. All the six inflammation indices were significantly higher in patients with LAT (Table 1).

**Table 1:** Baseline characteristics of the study population stratified by left atrial thrombus

	Total (n = 434)	No LAT (n = 291)	LAT (n = 143)	*P* value
Male, n (%)	157 (36.2%)	94 (32.3%)	63 (44.1%)	0.017
Age (years)	56.94 ± 9.12	57.16 ± 8.95	56.48 ± 9.48	0.465
Paroxysmal atrial fibrillation, n (%)	45 (10.4%)	34 (11.7%)	11 (7.7%)	0.200
First-diagnosed atrial fibrillation, n (%)	261 (60.1%)	168 (57.7%)	93 (65.0%)	0.174
Moderate/severe mitral stenosis	419 (96.5%)	279 (95.9%)	140 (97.9%)	0.404
Coronary heart diseases, n (%)	20 (4.6%)	14 (4.8%)	6 (4.2%)	0.774
Heart failure, n (%)	287 (66.1%)	182 (62.5%)	105 (73.4%)	0.024
Hypertension, n (%)	63 (14.5%)	46 (15.8%)	17 (11.9%)	0.276
Diabetes, n (%)	28 (6.5%)	13 (4.5%)	15 (10.5%)	0.016
Stroke, n (%)	79 (18.2%)	48 (16.5%)	31 (21.7%)	0.188
Oral anticoagulant, n (%)	142 (32.7%)	101 (34.7%)	41 (28.7%)	0.208
Beta-blockers, n (%)	76 (17.5%)	54 (18.6%)	22 (15.4%)	0.414
Renin-angiotensin system inhibitors, n (%)	24 (5.5%)	15 (5.2%)	9 (6.3%)	0.626
Spironolactone, n (%)	57 (13.1%)	34 (11.7%)	23 (16.1%)	0.202
Left atrium diameter (mm)	55.01 ± 11.35	54.09 ± 11.02	56.85 ± 11.38	0.022
Left ventricular ejection fraction (%)	56.92 ± 7.89	57.44 ± 7.89	55.89 ± 7.80	0.079
Estimated glomerular filtration rate (mL/min/1.73 m^2^)	88.51 ± 17.80	88.90 ± 17.29	87.72 ± 18.82	0.516
C-reactive protein (mg/L)	1.76 (0.65, 5.18)	1.20 (0.58, 2.97)	3.56 (1.38, 7.52)	0.001
White blood cell count (× 10^9^/L)	6.20 ± 2.08	6.08 ± 2.07	6.45 ± 2.11	0.082
Neutrophil count (× 10^9^/L)	3.91 ± 1.88	3.77 ± 1.84	4.19 ± 1.93	0.029
Lymphocytes count (× 10^9^/L)	1.69 ± 0.57	1.73 ± 0.56	1.62 ± 0.60	0.059
Monocyte count (× 10^9^/L)	0.43 ± 0.16	0.41 ± 0.16	0.46 ± 0.16	0.001
Platelet count (× 10^9^/L)	190.07 ± 57.32	192.46 ± 55.73	185.22 ± 60.33	0.217
Mean platelet volume (fL)	10.54 ± 1.32	10.61 ± 1.37	10.39 ± 1.22	0.100
NLR	2.64 ± 1.99	2.46 ± 1.80	3.00 ± 2.29	0.007
MLR	0.28 ± 0.16	0.26 ± 0.14	0.32 ± 0.18	< 0.001
WMR	0.60 ± 0.21	0.58 ± 0.21	0.63 ± 0.23	0.038
NMR	0.38 ± 0.19	0.36 ± 0.18	0.41 ± 0.20	0.014
SII	503.12 ± 431.26	471.17 ± 354.40	568.57 ± 551.60	0.027
SIRI	1.22 ± 1.38	1.10 ± 1.28	1.48 ± 1.52	0.007

*LAT* left atrial thrombus, *NLR* neutrophil/lymphocyte ratio, *MLR* monocyte/lymphocyte ratio, *WMR* white blood/mean platelet volume ratio, *NMR* neutrophil/mean platelet volume ratio, *SII* systemic immune inflammation index, *SIRI* system inflammation response index

### Relationship between inflammation indices and CRP

**Fig. 2:**
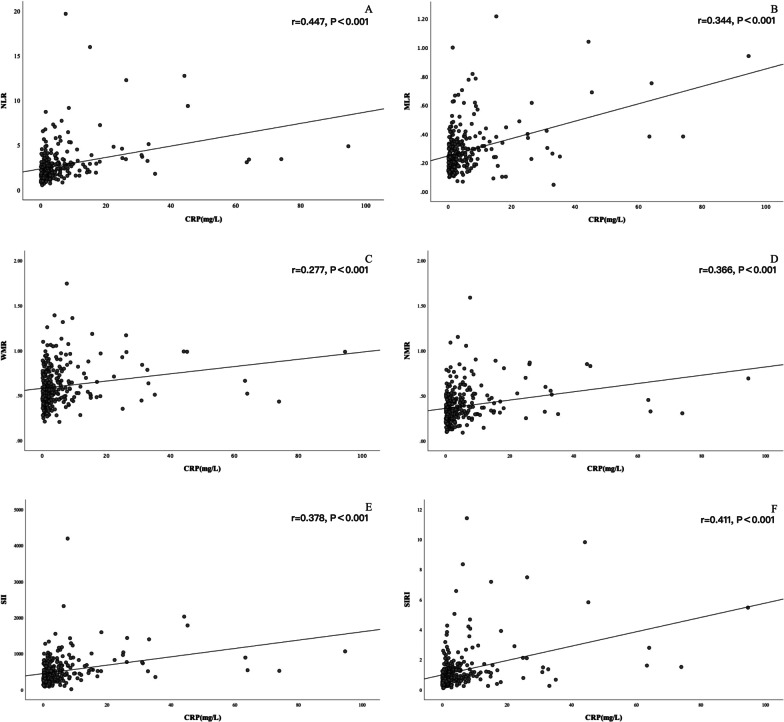
Correlations between inflammation indices and CRP. *NLR* neutrophil/lymphocyte ratio, *MLR* monocyte/lymphocyte ratio, *WMR* white blood/mean platelet volume ratio, *NMR* neutrophil/mean platelet volume ratio, *SII* systemic immune inflammation index, *SIRI* system inflammation response index, *CRP* C-reactive protein

In correlation analysis, the six inflammation indices were all positively correlated with CRP levels (*P* < 0.001, Fig. 2). NLR had the highest correlation coefficient (r = 0.447), whereas WMR had the lowest (r = 0.277).

### Dose–response association between inflammation indices and risk for LAT

**Fig. 3:**
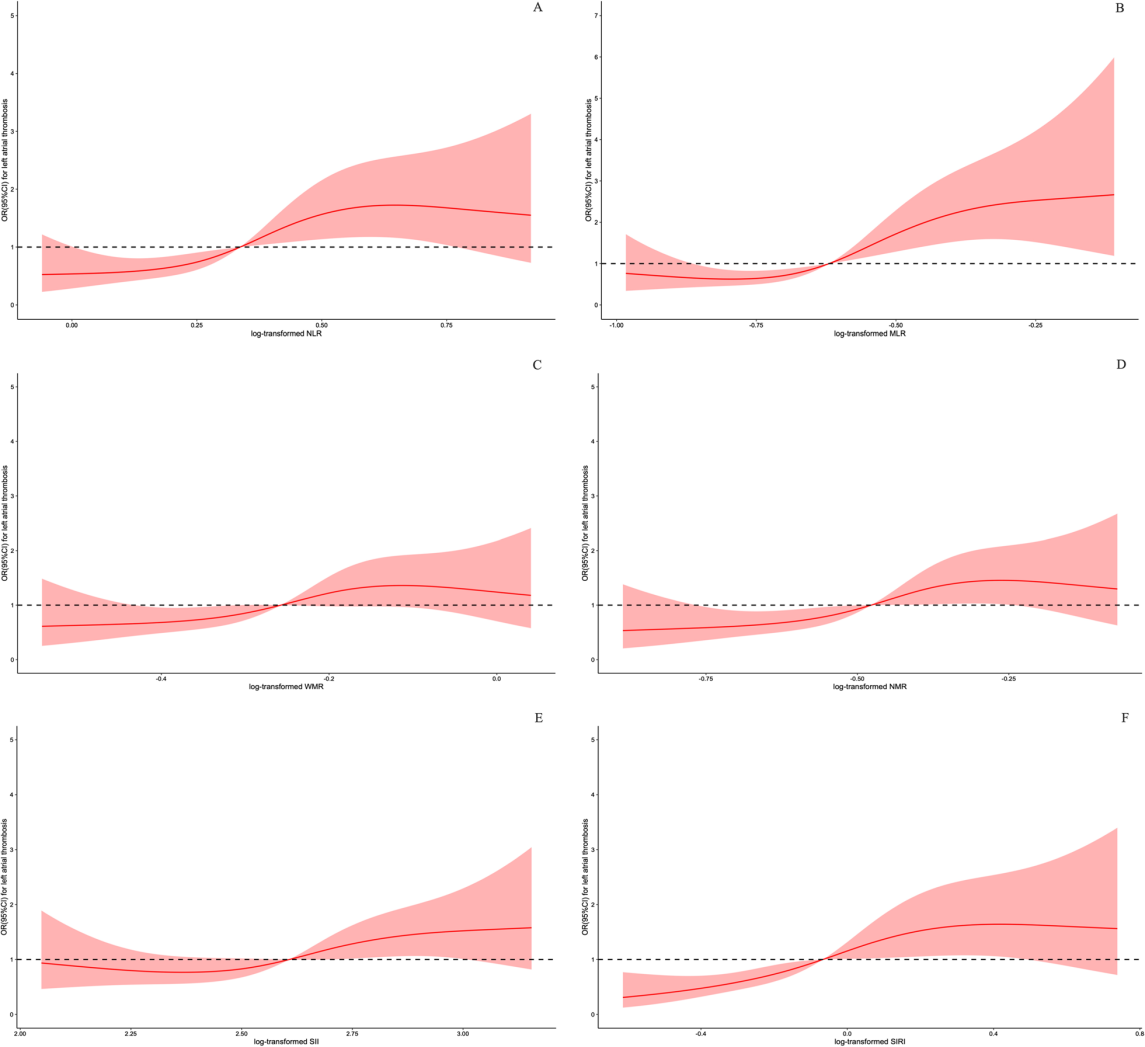
Dose–response curve of inflammation indices and risk of left atrial thrombus. *NLR* neutrophil/lymphocyte ratio, *MLR* monocyte/lymphocyte ratio, *WMR* white blood/mean platelet volume ratio, *NMR* neutrophil/mean platelet volume ratio, *SII* systemic immune inflammation index, *SIRI* system inflammation response index, *OR* odds ratio, *CI* confidence interval

To continuously assess the association of inflammation indices with the risk of LAT, dose–response curves were constructed (Fig. 3). Log-transformed NLR (*P* for non-linearity < 0.001), MLR (*P* for non-linearity < 0.001), NMR (*P* for non-linearity = 0.003) and SIRI (*P* for non-linearity < 0.001) all had a non-linear and positive correlation with the risk of LAT. The risk increased when the log-transformed NLR, MLR, NMR and SIRI were greater than 0.338, 0.619, 0.477 and 0.064, respectively. Linear and positive associations were found between log-transformed WMR (*P* for non-linearity = 0.129), SII (P for non-linearity = 0.126), and the risk of LAT. The risk increased when the log-transformed WMR and SII were above 0.255 and 2.610, respectively.

### Logistic regression analysis of the association between inflammation indices and LAT

Considering the possible covariance between the inflammation indices, separate logistic regression analyses were performed. As shown in Table 2, unadjusted logistic regression analyses revealed that all the six inflammation indices were associated with an increased rate of LAT. After adjusting for age and gender (model 1), inflammation indices maintained a significant association with LAT. The strength of this association was not attenuated after additional adjustment for thrombotic factors (coronary heart diseases, heart failure, hypertension, diabetes, and stroke; model 2). Finally, the significant association of all the six inflammation indices with the risk of LAT were still consistent after further adjustment for oral anticoagulants, LAD and LVEF (model 3).

**Table 2:** Logistics regression analysis to examine the association between inflammation indices and LAT

	Unadjusted model OR (95%CI)	*P* value	Model 1 OR (95%CI)	*P* value	Model 2 OR (95%CI)	*P* value	Model 3 OR (95%CI)	*P* value
NLR	4.482 (1.896–10.598)	0.001	4.369 (1.829–10.437)	0.001	4.794 (1.970–11.668)	0.001	4.324 (1.589–11.765)	0.004
MLR	9.205 (3.346–25.330)	< 0.001	9.406 (3.215–25.454)	< 0.001	9.978 (3.465–28.733)	< 0.001	12.006 (3.404–42.347)	< 0.001
WMR	4.908 (1.246–19.337)	0.023	4.383 (1.091–17.608)	0.037	7.421 (1.550–35.538)	0.012	6.736 (1.388–32.695)	0.018
NMR	4.356 (1.551–12.231)	0.005	4.096 (1.439–11.657)	0.008	4.116 (1.409–12.024)	0.010	4.953 (1.506–16.287)	0.008
SII	2.077 (1.016–4.248)	0.045	2.196 (1.069–4.509)	0.032	2.484 (1.192–5.175)	0.015	2.447 (1.081–5.538)	0.032
SIRI	3.717 (1.982–6.970)	< 0.001	3.579 (1.889–6.782)	< 0.001	3.713 (1.932–7.136)	< 0.001	3.988 (1.870–8.503)	< 0.001

Model 1: Adjusted for age and gender

Model 2: Additionally adjusted for thrombotic factors (Coronary heart diseases, Heart failure, Hypertension, Diabetes, Stroke)

Model 3: Further adjusted for Oral anticoagulant, Left atrial diameter and LVEF

All inflammation indices were logarithmically transformed

*NLR* neutrophil/lymphocyte ratio, *MLR* monocyte/lymphocyte ratio, *WMR* white blood/mean platelet volume ratio, *NMR* neutrophil/mean platelet volume ratio, *SII* systemic immune inflammation index, *SIRI* system inflammation response index, *LAT* left atrial thrombus, *LVEF* left ventricular ejection fraction, *OR* odds ratio, *CI* confidence interval

### Discriminative ability of inflammation indices

**Fig. 4:**
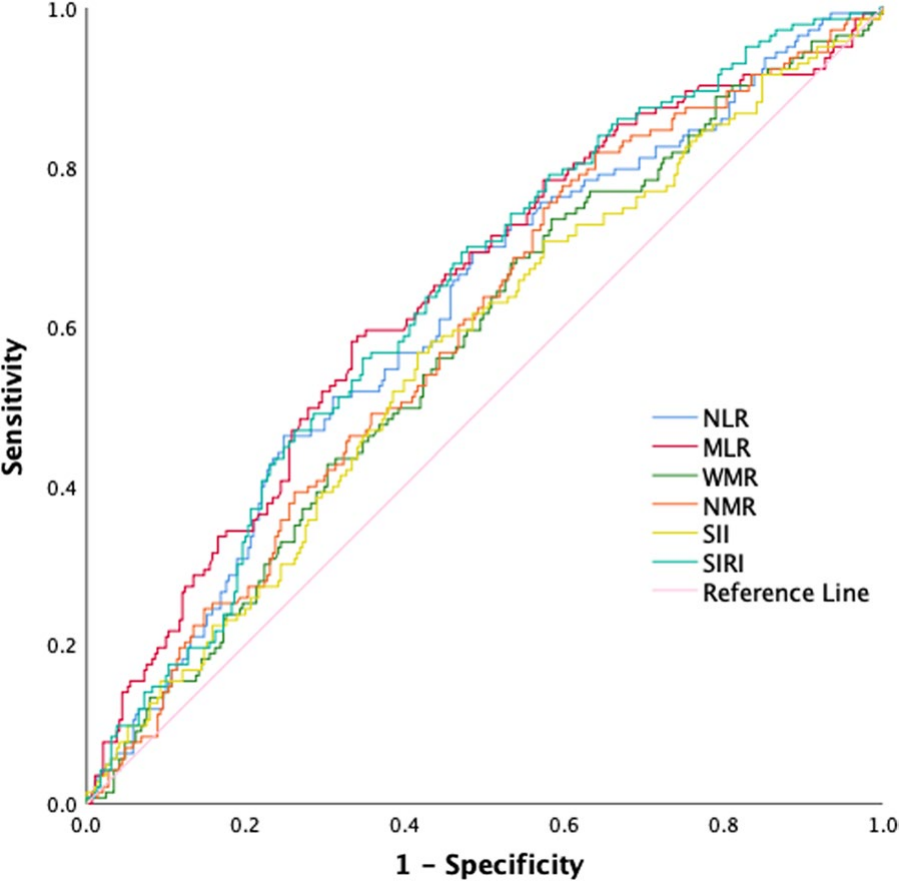
Receiver operating characteristic curves for inflammation indices as a predictor of left atrial thrombus. *NLR* neutrophil/lymphocyte ratio, *MLR* monocyte/lymphocyte ratio, *WMR* white blood/mean platelet volume ratio, *NMR* neutrophil/mean platelet volume ratio, *SII* systemic immune inflammation index, *SIRI* system inflammation response index

The comparisons among various inflammation indices for predicting LAT were summarized in Table 3. MLR had the highest AUC (0.639 [95% CI 0.583–0.694]; *P* < 0.001). The ROC curves for inflammation indices were presented in Fig. 4. According to pair-wise comparison of the AUCs, MLR appeared to perform better than the other five indices (Table 4).

**Table 3:** Discrimination ability of inflammatory indices

	NLR	MLR	WMR	NMR	SII	SIRI
Cutoff value	2.663	0.261	0.497	0.272	423.334	0.789
Sensitivity (%)	46.2	58.0	73.4	81.8	56.6	69.2
Specificity (%)	75.3	66.7	41.6	36.1	58.4	52.9
AUC (95% CI)	0.614 (0.558–0.670)	0.639 (0.583–0.694)	0.574 (0.518–0.630)	0.591 (0.535–0.647)	0.568 (0.510–0.625)	0.635 (0.581–0.689)
*P* value	< 0.001	< 0.001	0.012	0.002	0.022	< 0.001

*NLR* neutrophil/lymphocyte ratio, *MLR* monocyte/lymphocyte ratio, *WMR* white blood/mean platelet volume ratio, *NMR* neutrophil/mean platelet volume ratio, *SII* systemic immune inflammation index, *SIRI* system inflammation response index, *AUC* area under the curve, *CI* confidence interval

**Table 4:** The pair-wise comparison of AUCs among inflammation indices

Comparison	AUC	
	Difference (95% CI)	*P* value
NLR–MLR	− 0.025 (− 0.073, 0.023)	0.313
NLR–WMR	0.040 (− 0.022, 0.101)	0.205
NLR–NMR	0.023 (− 0.022, 0.067)	0.319
NLR–SII	0.046 (0.015, 0.077)	0.004
NLR–SIRI	− 0.021 (− 0.055, 0.013)	0.226
MLR–WMR	0.065 (− 0.01, 0.139)	0.089
MLR–NMR	0.048 (− 0.02, 0.115)	0.167
MLR–SII	0.071 (0.015, 0.127)	0.014
MLR–SIRI	0.004 (− 0.031, 0.039)	0.824
WMR–NMR	− 0.017 (− 0.038, 0.004)	0.120
WMR–SII	0.006 (− 0.049, 0.062)	0.822
WMR–SIRI	− 0.061 (− 0.111, − 0.010)	0.018
NMR–SII	0.023 (− 0.019, 0.065)	0.275
NMR–SIRI	− 0.044 (− 0.083, − 0.004)	0.032
SII–SIRI	− 0.067 (− 0.108, − 0.026)	0.001

*NLR* neutrophil/lymphocyte ratio, *MLR* monocyte/lymphocyte ratio, *WMR* white blood/mean platelet volume ratio, *NMR* neutrophil/mean platelet volume ratio, *SII* systemic immune inflammation index, *SIRI* system inflammation response index, *AUC* area under the curve, *CI* confidence interval

## Discussion

To our knowledge, the present study was the first to examine the predictive value of inflammation indices for LAT in patients with VAF. Our principal finding was that elevated inflammation indices were robustly associated with an increased risk for LAT. These indices could reveal the level of systemic inflammation due to their positive correlation with conventional inflammation biomarkers such as CRP. Systemic inflammation was one of the common determinants of VAF and thrombogenesis. Among the six inflammation indices, MLR yielded the highest AUC (0.639), confirming its predictive value. Additionally, the inflammation indices are extensively and readily obtainable at low cost in the laboratory and clinical fields, and VAF is largely limited to low- and middle- income countries, so these indices may be proposed as predictors for the incidence of LAT.

To date, there has been a paucity of information regarding the association between inflammation indices and LAT or stroke in patients with AF. These studies were limited to NVAF patients and the index of NLR. A study including 207 NVAF patients demonstrated that NLR was significantly and positively correlated with the CHA_2_DS_2_-VASc score and CRP, and a higher NLR was associated with a 3.872-fold risk for LAT [12]. Yalcin et al. [13] found a similar significant association in a larger sample, and reported that a higher NLR was an independent risk factor for LAT in 309 patients with NVAF (OR 1.59). Fukuda et al. [14] demonstrated that an elevated NLR was an independent risk factor for spontaneous echo contrast in TEE (OR 1.86) and had greater left atrial volume index, left atrial appendage area and left atrial appendage wall motion velocity during atrial contraction. This study revealed an association between NLR and the left atrial appendage function in relation to thrombogenesis. In a cohort study involving 981 patients with AF, NLR was also significantly associated with the first episode of stroke independent of the CHA_2_DS_2_-VASc score and this association had a dose–response pattern [15]. Similar to these previous studies, our study verified the predictive value of NLR for LAT formation in VAF patients. Moreover, we found that other inflammation indices were also independent risk factors for LAT and some were not worse than the NLR. Inflammation indices can be mutually complementary. NLR had a poor sensitivity for LAT (46.2%), while NMR had the best sensitivity (81.8%). A combination of inflammation indices may improve the accuracy of predicting LAT.

The presence of systemic inflammation in VAF patients was found by either histopathological examination of the left atrium or by measuring serum inflammatory biomarkers. In local cardiac tissue, VAF patients exhibited higher infiltration of neutrophils and resting stage dendritic cells, while those with sinus rhythm exhibited higher infiltration of follicular helper T cells [16]. Neutrophil-mediated inflammatory responses were mainly associated with neutrophil extracellular traps that recruited other inflammatory cells such as macrophages to amplify the inflammatory response and promoted collagen synthesis leading to fibrosis. Sharma et al. [17] found a progressive increase in the level of inflammatory biomarkers (CRP, interleukin‐6 and sCD‐40L) in rheumatic mitral stenosis patients with sinus rhythm, subclinical transient AF and chronic AF. CRP, interleukin‐6 and sCD‐40L had also been recognized as predictors of thromboembolic complications in AF patients in several studies [8, 18, 19]. Inflammatory cytokines had prothrombotic effects such as upregulation of tissue factors from monocyte-macrophages, increased fibrinogen expression, reduced expression of protein C and related proteins, and increased platelet reactivity. Inflammation also caused and accelerated the electrical and structural remodeling of the atria, resulting in ineffective irregular contraction and blood stasis. Blood stasis in the left atrium played a vital role in thrombogenic tendency among patients with AF. During inflammatory reactions, blood cell subtype counts and the balance between them changed. A combination of these cell counts might better reflect alterations in systemic inflammation. Inflammation indices in our study were all positively correlated with CRP; therefore, their levels also reflected the extent of systemic inflammation. Furthermore, it was reported that the NLR could be reduced by anti-inflammatory therapy using canakinumab [20]. Another promising pharmacological agent, sodium-glucose cotransporter 2 inhibitor, could also decrease the level of NLR by directly targeting inflammatory pathways such as nucleotide-binding domain-like receptor protein-3 inflammasome [21, 22]. Recent evidence suggested that inflammation indices could identify high-risk patients with thromboembolic diseases such as acute pulmonary embolism or peripheral arterial disease [23, 24]. Therefore, based on our study and findings from previous investigations, systemic inflammation might underlie the association between the indices examined in our study and LAT in VAF patients.

Our study had several limitations, the first of which was its retrospective design, which was not specifically constructed to assess the endpoints reported in this article. As such, prospective studies with larger sample sizes are required to confirm our results. In addition, many factors, such as smoking, alcohol, and mental stress, can induce chronic systemic inflammation; however, these data were not collected in our study. Finally, inflammatory indices were measured once at admission and subsequent temporal changes were not observed. Dynamic monitoring of these indices may provide additional information.

## Conclusion

Elevated inflammatory indices were associated with an increased risk for LAT in patients with VAF. Because these indices are extensively used and readily available in the clinical field, we propose that they could be used as cost-effective predictors for thromboembolic risk, which would benefit a large subset of patients with VAF in developing countries.

## Data Availability

The datasets generated and analysed during the current study are not publicly available due to the Henan Provincial People’s Hospital regulations, but are available from the corresponding author on reasonable request.

## Abbreviations

AF: Atrial fibrillation
NVAF: Non-valvular atrial fibrillation
VAF: Valvular atrial fibrillation
LAT: Left atrial thrombus
CRP: C-reactive protein
NLR: Neutrophil-to-lymphocyte ratio
MLR: Monocytes-to-lymphocyte ratio
WMR: White blood cell-to-mean platelet volume ratio
NMR: Neutrophil-to-mean platelet volume ratio
SII: Systemic immune inflammation index
SIRI: System inflammation response index
TEE: Transesophageal echocardiography
LAD: Left atrium diameter
LVEF: Left ventricular ejection fraction
ROC: Receiver operating characteristic curve
AUC: Area under the curve
OR: Odds ratio
CI: Confidence interval
